# Meningeal enhancement following traumatic brain injury: a mini review

**DOI:** 10.3389/fneur.2025.1617126

**Published:** 2025-07-14

**Authors:** Alejandro Arbona-Lampaya, Alejandro Odeh-Couvertier, Ricardo Sánchez Jiménez, Eduardo Labat Álvarez

**Affiliations:** ^1^School of Medicine, University of Puerto Rico–Medical Sciences Campus, San Juan, Puerto Rico; ^2^Department of Diagnostic Radiology, University of Puerto Rico School of Medicine, San Juan, Puerto Rico

**Keywords:** traumatic meningeal enhancement, traumatic brain injury, subdural hematoma, blood–brain barrier disruption, imaging biomarkers

## Abstract

Traumatic brain injury (TBI) is a significant cause of neurological morbidity, often leading to blood–brain barrier (BBB) dysfunction and secondary injury mechanisms. Recent advancements in neuroimaging have highlighted traumatic meningeal enhancement (TME) on contrast-enhanced fluid-attenuated inversion recovery (FLAIR) MRI as a promising biomarker for detecting BBB disruption following TBI. TME, which is hypothesized to arise from vascular injury and inflammatory cascades that compromise the blood-meningeal barrier, has been associated with both acute trauma and long-term neurovascular dysfunction. Its presence, particularly when linked to subdural hematomas and delayed contrast extravasation, not only reflects the immediate severity of the injury but may also indicate chronic neuroinflammatory processes and persistent cognitive deficits. In this review, we gather current evidence on the pathophysiology of TME including its associations with vascular permeability, subdural hematoma, and prolonged inflammatory responses. We explore its potential as a biomarker for injury severity and prognosis in TBI patients. Finally, we further discuss the critical need for standardized imaging protocols and longitudinal studies to determine the clinical implications of persistent TME.

## Graphical Abstract

**Figure:**
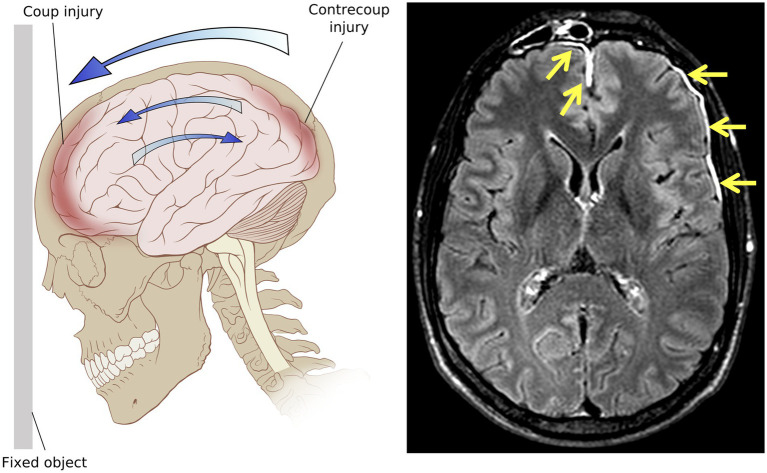
Left panel: “Contrecoup” by Patrick J. Lynch, medical illustrator; C. Carl Jaffe, MD, cardiologist is licensed under CC BY 2.5. Right panel: Adapted with permission from “Subdural hematoma and traumatic meningeal enhancement in patients with acute traumatic brain injury” by Arbona-Lampaya et al. (15).

## 1 Introduction

Traumatic brain injury (TBI) is a neuropsychiatric disorder that results from sudden, external, physical damage to the brain (1). It is estimated that each year 69 million people suffer a TBI worldwide (2). TBI can result in temporary or short-term problems with brain function, while more serious TBI can lead to severe and permanent disability, or even death. Mild TBI (mTBI) is defined clinically as one in which the duration of unconsciousness is less than 30 minutes, the duration of posttraumatic amnesia is less than 24 hours, and the Glasgow Coma Scale (GCS) score is 13 to 15 out of a possible 15 (3). Computed tomography (CT) scans are the most often used imaging technique in the diagnosis of TBI due to its general availability. However, only 5–10% of mTBI patients with GCS scores of 15 have abnormal CT scans (3). Chiara et al. (4) demonstrated the feasibility of utilizing a fast magnetic resonance imaging (MRI) protocol on TBI patients in identifying trauma-related abnormalities not identified on CT.

**Figure 1:**
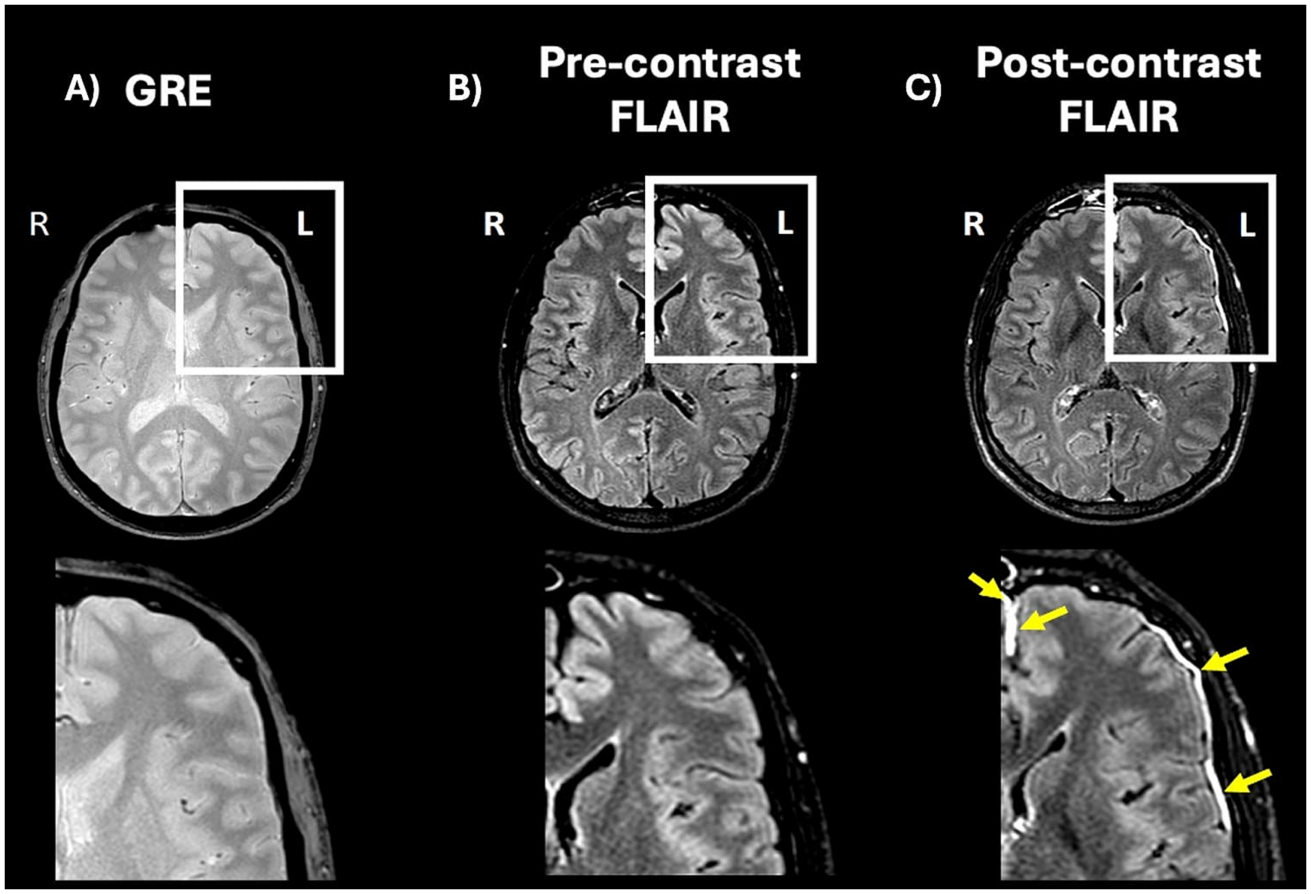
Axial MRI scans from a patient with traumatic brain injury demonstrating the absence of subdural hematoma (SDH) but presence of traumatic meningeal enhancement (TME). **(A)** Gradient echo (GRE) sequence shows no evidence of hemorrhage. **(B)** Pre-contrast fluid attenuated inversion recovery (FLAIR) sequence shows no abnormal meningeal signal. **(C)** Post-contrast FLAIR sequence reveals hyperintense enhancement along the left convexity (yellow arrows), consistent with TME in the absence of visible SDH. These findings highlight that TME can occur independently of hemorrhage and may represent isolated meningeal injury or subtle blood–brain barrier disruption. Adapted from Arbona-Lampaya et al. (15).

Traumatic meningeal enhancement (TME) (Figure 1) is an MRI finding increasingly recognized in patients with TBI and may have diagnostic and prognostic significance. It can be observed on post-contrast fluid-attenuated inversion recovery (FLAIR) in patients who undergo contrast-enhanced MRI after suspected TBI (5). It refers to the observable enhancement of the meninges on MRI scans following TBI. This enhancement occurs due to the disruption of the blood–brain barrier (BBB) or blood-meningeal barrier (BMB), allowing contrast agents to penetrate the meninges after trauma (6, 7). It has been shown in the literature that TME can be detected on contrast-enhanced MRI in patients presenting with acute TBI, even when head CT is negative (5–7). TME varies in its radiological appearance, ranging from subtle linear enhancement to more conspicuous nodular or sheet-like patterns. Turtzo et al. (7) described this variability across patients and imaging time points, suggesting that the degree of enhancement may reflect underlying biological heterogeneity in injury severity or healing response. The recent increase in recognition of TME is not due to technological innovation, but rather to the more frequent inclusion of contrast-enhanced FLAIR sequences in contrast-enhanced imaging protocols.

Anatomically, TME is most frequently observed in the dura mater and dural border cell layer, making it primarily a pachymeningeal phenomenon. Studies by Kim et al. (8) and Turtzo et al. (7) reported enhancement in locations such as the falx cerebri, tentorium, and convexity dura, areas rich in dural venous sinuses and vasculature, which are particularly vulnerable to traumatic shear forces. Turtzo et al. (7) further proposed that gadolinium-based contrast may accumulate in cerebrospinal fluid (CSF) like that collects between the dura and arachnoid following injury, potentially creating a space of enhancement. While less common, limited enhancement suggestive of leptomeningeal involvement has been described, but its interpretation remains uncertain and may reflect secondary spread or more severe injury.

Notably, while post-contrast T1-weighted imaging (T1WI) is more routinely used in clinical practice, post-contrast FLAIR imaging has been shown to have significantly greater sensitivity and inter-rater reliability for detecting TME. In a head-to-head comparison, Davis et al. (5) demonstrated that 38% of TME-positive cases seen on post-contrast FLAIR were not visible on post-contrast T1WI, and interrater agreement was far superior for FLAIR (*κ* = 0.90 vs. κ = −0.24). This superiority is attributed to FLAIR’s enhanced sensitivity to contrast within CSF-like spaces, where TME is believed to occur due to BMB disruption.

In this review, we will explore the pathophysiology of TME, its potential as a biomarker for TBI severity, and the current gaps in research that warrant further investigation. Understanding TME and its implications can guide future research into the mechanisms of brain injury and recovery, potentially leading to improved treatment strategies for TBI patients.

## 2 Traumatic meningeal enhancement

### 2.1 Pathophysiology of traumatic meningeal enhancement

#### 2.1.1 Blood–brain barrier vs. blood-meningeal barrier disruption

The pathophysiological basis of TME remains incompletely understood, with ongoing debate about whether TME primarily reflects vascular injury, inflammation, or a combination of both. The BBB is composed of endothelial cells connected by tight junctions and surrounded by pericytes and astrocyte end-feet, regulating solute exchange between the blood and the brain parenchyma. In contrast, the BMB refers to the vascular interfaces of the dura mater and leptomeninges, which are structurally distinct from the BBB and lack the same tight junction complexity. The BMB includes dural venous sinuses, meningeal capillaries, and associated immune interfaces that separate blood from cerebrospinal fluid and meningeal tissue (9, 10). Due to these anatomical and functional differences, findings from BBB studies cannot be universally extrapolated to the BMB without specific supporting evidence.

TME is linked to increased vascular permeability, allowing gadolinium contrast leakage into the meningeal space, as visualized on post-contrast MRI. This phenomenon results from disruption of the BMB. Following TBI, mechanical forces and subsequent inflammatory cascades compromise BMB integrity, leading to abnormal permeability and contrast extravasation (6, 9). Supporting evidence for vascular injury comes from dynamic contrast-enhanced MRI (DCE-MRI) studies by Castro et al. (11), who observed increased permeability in regions of TME compared to unaffected tissue, consistent with a breakdown of normal vascular integrity. Roth et al. (12) corroborated this in an animal model, showing reactive oxygen species-mediated vascular damage and BBB leakage following TBI. Turtzo et al. (7) reinforced this hypothesis, showing a high prevalence (53%) of acute TME, highlighting its consistent association with abnormal extravasation of contrast into the meningeal spaces in acute TBI patients.

Evidence for an inflammatory mechanism has also been substantiated. McNamara et al. (10), using the CHIMERA mouse model, observed that traumatic impact led to persistent enhancement on MRI alongside upregulation of pro-inflammatory genes and disruption of astrocytic tight junctions. Mokbel et al. (9) further supported this by identifying significant infiltration of immune cells, particularly CD45 + leukocytes, within the meninges after TBI, as well as dysregulation of lymphatic clearance, contributing to prolonged inflammation. Turtzo et al. (6) found elevated levels of inflammatory cytokines such as IL-6 and MCP-1 in CSF of TME-positive patients, suggesting a direct correlation between enhancement and neuroinflammatory signaling.

Castro et al. (11) suggested that BBB disruption contributes significantly to TME development, based on findings of increased vascular permeability in affected regions, although without definitive conclusions on inflammatory contributions. Similarly, Liraz-Zaltsman et al. (13) demonstrated prolonged vascular permeability in adjacent brain tissue using DCE-MRI, reinforcing the idea that BBB involvement cannot be entirely excluded, even when the primary site of injury appears meningeal. While Mokbel et al. (9) emphasize the role of meningeal immune activation, others, including Turtzo et al. (20) and Liraz-Zaltsman et al. (13), provide evidence that TME results from sustained BBB permeability changes. Turtzo et al. (7) builds upon this foundation by demonstrating both the prevalence and clinical impact of persistent TME, reaffirming that it likely reflects a complex interplay of ongoing neurovascular injury and inflammation.

There remains ongoing debate as to whether TME primarily reflects disruption of the BBB, the BMB, or both. While many early studies refer broadly to BBB dysfunction, more recent work suggests that TME is anatomically and physiologically distinct from classic parenchymal BBB injury. Given that TME typically localizes to the dura or dural border cell layer, regions not protected by the parenchymal BBB, it is more likely to reflect injury to the BMB, which governs solute exchange in the meninges and is structurally less restrictive. Although the BMB and BBB are anatomically and functionally distinct, they are closely aligned, and injury to the BMB may reflect or even contribute to adjacent BBB disruption. Multiple studies have independently reported increased permeability in either the BBB or the BMB, suggesting that TME may broadly reflect neurovascular injury across both compartments (6, 7, 11, 13).

#### 2.1.2 Association with subdural hematoma

Subdural hematoma (SDH) is one of the most common types of intracranial hemorrhage observed after TBI and is present across the full spectrum of severity, including both mild and severe TBI. They account for 66–75% of all neurosurgical procedures, and they have one of the highest neurosurgical intervention rates (14). Studies have documented a robust association between traumatic meningeal TME and SDH, implying shared underlying mechanisms involving meningeal and vascular disruption. Kim et al. (8) initially highlighted this correlation, demonstrating that TME detected by contrast-enhanced FLAIR imaging frequently co-occurs with SDH, reflecting vascular injury and meningeal compromise.

Roth et al. (12) added mechanistic evidence through animal models, showing that vascular damage and reactive oxygen species generation in the meninges precede the development of TME-like findings. Their results show that traumatic injury could result in both meningeal vascular disruption visible as SDH and BBB compromise leading to TME, reinforcing the hypothesis that SDH and TME share a common pathophysiological mechanism. Castro et al. (11) further reinforced this connection, suggesting substantial overlap between TME and SDH on DCE-MRI, likely due to shared traumatic vascular pathology. Their results indicate that TME and SDH may be different manifestations of the same underlying vascular disruption, which could explain why TME is frequently observed in patients with SDH.

Studies have shown that SDH is associated with TME, but the location of TME can be widespread throughout the meninges without a clear pattern (6, 7). Arbona-Lampaya et al. (15) found that SDH and TME were observed together in 76 of 77 patients, strongly supporting the hypothesis of a common underlying pathology. They noted that isolated SDH without concurrent TME was extremely rare, implying that TME may be a sensitive marker for detecting subtle vascular and meningeal injury even when SDH is clinically unapparent. Similarly, Turtzo et al. (7) confirmed the significant association between TME and intracranial hemorrhage, including SDH, but notably also identified instances of TME in patients without visible SDH, underscoring the possibility of TME reflecting a broader spectrum of meningeal injury beyond overt hemorrhage. This finding is consistent with Arbona-Lampaya et al. (15), who observed that TME could occur independently of SDH, potentially representing milder or subclinical meningeal damage.

However, the co-occurrence of TME and SDH alone does not confirm a unique traumatic pathophysiology since non-traumatic chronic SDH can also show meningeal enhancement. Notably, Hassan et al. (16) described the meningeal enhancement associated with chronic SDH as typically involving consistent, localized enhancement of the outer membrane adjacent to the cranium, occasionally extending to the inner membrane. This is distinct from the pattern observed in traumatic cases, where TME often manifests as diffuse, widespread enhancement. This pattern seen in traumatic cases could reflect acute and incomplete meningeal repair processes (7). Additionally, meningeal enhancement associated with non-traumatic SDH typically occurs in the subacute to chronic timeframe, compared to TME which occurs in the acute timeframe (5, 7, 16). Furthermore, traumatic meningeal enhancement detected via contrast-enhanced FLAIR imaging is more extensive and diffuse, frequently correlating with acute SDH and loss of consciousness (8).

Kim et al. (8) documented the association of TME with subarachnoid hemorrhage (SAH). Their study showed that meningeal enhancement can occur due to blood entering the CSF spaces after traumatic SAH, reflecting injury-induced inflammation and increased vascular permeability of the meningeal barriers. Turtzo et al. (6) noted that traumatic damage to the arachnoid membrane can result in contrast extravasation from areas of TME into the adjacent subarachnoid space, manifesting as extra-axial contrast-enhanced subarachnoid spaces, indicating that TME can co-occur or extend into areas typically involved in traumatic SAH. Turtzo et al. (7) showed statistically significant co-occurrence between TME and SAH (*p* = 0.008). Thus, beyond SDH, TME’s relationship with other types of brain hemorrhages such as SAH is recognized, particularly where damage extends into subarachnoid spaces though there are few studies exploring this relationship.

#### 2.1.3 Delayed contrast extravasation and meningeal injury

Delayed contrast enhancement on post-contrast FLAIR MRI has emerged as a key imaging feature of TME, reflecting prolonged BBB and BMB dysfunction after TBI. In this context, “delayed” refers to the slow kinetics of gadolinium accumulation within the meningeal space after contrast administration, not merely the timing of MRI acquisition days or weeks after trauma. Studies using DCE-MRI, such as those by Liraz-Zaltsman et al. (13), do not directly evaluate TME but provide indirect support for this mechanism by modeling subtle, prolonged BBB disruption in mouse models of TBI. In that study, delayed contrast extravasation MRI revealed persistent gadolinium leakage for up to 540 days post-injury, which was not detected on standard T1-weighted or T2-weighted MRI. This enhancement was histologically validated with ZO-1 tight junction breakdown, reduced astrocytic vascular coverage, and IgG leakage, all supporting chronic microvascular dysfunction.

These findings were similar to those in Roth et al. (12) which used a mouse model of mild closed-skull TBI and observed vascular leakage of quantum dots and contrast agents into the meninges shortly after injury. They also identified reactive oxygen species and astrocytic damage, which collectively compromised the glial limitans, the barrier between meninges and brain parenchyma, leading to delayed parenchymal injury. Both studies describe a temporal sequence where vascular injury precedes and contributes to delayed imaging changes, in Roth et al. (12) via intravital two-photon laser scanning microscopy, and in Liraz-Zaltsman et al. (13) via delayed MRI enhancement.

Castro et al. (11) further demonstrated that regions of TME exhibit prolonged post-contrast signal on DCE-FLAIR, consistent with delayed gadolinium washout and persistent vascular permeability. Their findings suggest that the enhancement reflects contrast accumulation in fluid-filled compartments with slow clearance, particularly within the dura, falx, and tentorium.

Turtzo et al. (7) provide further mechanistic clarity by describing TME as the result of trauma-induced meningeal injury that permits the formation of newly developed fluid-filled spaces, likely between the dura and arachnoid membranes, into which gadolinium-based contrast agents extravasate. This leakage leads to shortening of the T1 relaxation time and visible hyperintensity on T2-FLAIR imaging. This conceptual model is based on the unique imaging behavior of contrast in CSF-like compartments, where gadolinium enhancement becomes more conspicuous on FLAIR due to suppressed background signal. While this explanation reinforces the role of contrast extravasation in TME and aligns with prior reports, such as Davis et al. (5), who emphasized the utility of post-contrast FLAIR in capturing subtle meningeal enhancement, it remains a theoretical framework. Turtzo et al. (7) note that direct histological validation of these spaces is lacking. Moreover, they raise an important mechanistic question that expands on previous literature: whether TME requires concomitant vascular damage or whether disruption of non-vascular barriers, such as the arachnoid membrane, is sufficient to allow contrast leakage. This nuanced view introduces the possibility that meningeal enhancement may result from a combination of mechanisms, including injury to the leptomeningeal vasculature and/or nonvascular meningeal membranes, and not solely from classic BBB disruption.

These findings not only support previous reports of delayed permeability changes but also deepen the understanding of TME pathophysiology by introducing the possibility that extravasation may occur not only through injured capillaries or venules but also due to disruption of adjacent meningeal barriers such as the arachnoid membrane. Collectively, they suggest that TME may represent a complex and prolonged meningeal healing response, emphasizing the importance of imaging timing and technique when assessing post-traumatic meningeal abnormalities.

### 2.2 Clinical implications of traumatic meningeal enhancement in traumatic brain injury

#### 2.2.1 Traumatic meningeal enhancement as a potential biomarker for traumatic brain injury severity

TME is observed in approximately 50–80% of TBI patients undergoing contrast-enhanced MRI, making it a relatively common finding (10, 20). Turtzo et al. (7) further solidified these estimates, identifying acute TME in approximately 53% of TBI patients within the first 48 h post-injury. Their findings highlighted acute TME’s high predictive value for associated acute TBI-related findings such as intracranial hemorrhage and parenchymal lesions on CT and MRI. Kim et al. (8) previously demonstrated that TME, detected by contrast-enhanced FLAIR imaging, correlates strongly with clinical presentations such as SDH and loss of consciousness, suggesting that TME presence aligns closely with clinically relevant injury severities.

McNamara et al. (10) demonstrated variability in TME prevalence depending on injury severity and frequency, indicating that TME may reflect a spectrum of injury severity. Turtzo et al. (7) similarly described TME as variably severe, grading enhancement from subtle to conspicuous, supporting the hypothesis that TME severity could mirror clinical and functional impairment. Mokbel et al. (9) emphasized that meningeal inflammation and BBB dysfunction are central to TBI pathology, proposing chronic meningeal immune activation as a contributor to cognitive impairment. These collective insights, strengthened by Turtzo et al. (7), support TME’s potential role as an imaging biomarker for evaluating TBI severity and prognosis.

#### 2.2.2 Persistent traumatic meningeal enhancement and long-term outcomes

Several studies indicate TME can persist beyond acute phases of TBI, correlating with chronic pathology and long-term outcomes. Rizk et al. (17) showed persistent TME in 17% of mild TBI patients and 63% in moderate to severe cases, extending to one year post-injury. Martinez et al. (18) further identified persistent TME two years post-injury. Adding to these findings, Turtzo et al. (7) provided robust evidence that persistent TME significantly correlates with poorer clinical recovery, defined by incomplete recovery on the Glasgow Outcome Scale—Extended (GOSE). Their study demonstrated that patients with persistent TME had nearly a fourfold higher likelihood of incomplete recovery compared to those without TME persistence, solidifying TME’s value as a prognostic marker.

These findings align well with Livingston et al. (19) and Mokbel et al. (9), who proposed persistent TME as indicative of ongoing inflammation and impaired lymphatic drainage, potentially underpinning delayed recovery and chronic symptoms. However, Turtzo et al. (7) made a novel, explicit claim regarding the clinical prognostic implications of persistent TME, providing substantial evidence to justify longitudinal imaging and clinical follow-up of TBI patients with initial TME findings.

### 2.3 Gaps in current research

Significant research gaps remain despite growing recognition of TME as a relevant marker of TBI pathology. Few studies have explored whether TME persists over extended periods or its exact correlation with chronic post-traumatic symptoms. Turtzo et al. (7) addressed part of this gap, demonstrating that persistent TME at 30–90 days is independently associated with incomplete clinical recovery, thus providing critical evidence supporting TME’s clinical relevance. Nonetheless, longitudinal studies extending beyond one year remain scarce and are crucial to understanding TME’s full prognostic implications (17, 18). Furthermore, variability in contrast administration, imaging sequences, and timing across studies, as highlighted by Castro et al. (11), limits comparability. Standardization of imaging protocols is thus necessary for consistent detection and clinical utility of TME.

Moreover, despite evidence linking TME to BBB dysfunction and inflammation (7, 9), the exact mechanisms, whether predominantly inflammatory or vascular, remain unresolved. Studies such as Mokbel et al. (9) and McNamara et al. (10) emphasize immune activation and vascular disruption localized to the meninges, supporting the notion that TME arises primarily from BMB injury. However, other studies, including Liraz-Zaltsman et al. (13) and Castro et al. (11), report prolonged vascular permeability in adjacent brain tissue using dynamic imaging techniques, suggesting that BBB involvement may also contribute. This demonstrates a fundamental gap in the literature, namely, the need to distinguish between BBB and BMB contributions to TME. Clarifying these mechanisms will enhance our understanding of TME’s clinical significance, improving patient stratification and targeted therapy in TBI management.

## 3 Conclusion

TME has emerged as a promising imaging biomarker that reflects underlying pathophysiological processes following TBI, particularly those related to BBB disruption, vascular injury, and meningeal inflammation. It is detectable even in patients with negative CT scans, expanding the diagnostic and prognostic landscape in both mild and moderate-to-severe TBI. Studies consistently show that TME is associated with clinical features such as SDH and loss of consciousness and may persist for months or even years post-injury, especially in patients with more severe trauma. Recent evidence, particularly from Turtzo et al. (7) note that direct histological validation of these spaces is lacking. Their findings demonstrate that persistent TME significantly increases the risk of incomplete recovery, suggesting it may capture ongoing neuroinflammatory or vascular dysfunction not otherwise visible on standard imaging. While much progress has been made, key knowledge gaps remain, particularly around the precise mechanisms that drive TME and the standardization of imaging protocols. Future research should prioritize longitudinal studies and multimodal imaging approaches to determine the full diagnostic and therapeutic utility of TME in the management of TBI.
